# Impact of Acoustic and Optical Phonons on the Anisotropic Heat Conduction in Novel C-Based Superlattices

**DOI:** 10.3390/ma17194894

**Published:** 2024-10-05

**Authors:** Devki N. Talwar, Piotr Becla

**Affiliations:** 1 Department of Physics, University of North Florida, 1 UNF Drive, Jacksonville, FL 32224, USA; 2 Department of Physics, Indiana University of Pennsylvania, 975 Oakland Avenue, 56 Weyandt Hall, Indiana, PA 15705, USA; 3 Department of Materials Science and Engineering, Massachusetts Institute of Technology, Cambridge, MA 02139, USA; becla@mit.edu

**Keywords:** novel (SiC)_m_/(GeC)_n_ superlattices, lattice dynamics, zone folding effect, anisotropy of phonons, phonon conductivity, thermal transport and management

## Abstract

C-based XC binary materials and their (XC)_m_/(YC)_n_ (X, Y ≡ Si, Ge and Sn) superlattices (SLs) have recently gained considerable interest as valuable alternatives to Si for designing and/or exploiting nanostructured electronic devices (NEDs) in the growing high-power application needs. In commercial NEDs, heat dissipation and thermal management have been and still are crucial issues. The concept of phonon engineering is important for manipulating thermal transport in low-dimensional heterostructures to study their lattice dynamical features. By adopting a realistic rigid-ion-model, we reported results of phonon dispersions $\omega_{j}^{SL}\left(\vec{k}\right)\;of\;novel\;short-period\;{\left(XC\right)}_{m}/{\left(YC\right)}_{n}\left[001\right]\;SLs$, for m, n = 2, 3, 4 by varying phonon wavevectors $\left|{\vec{k}}_{SL}\right|$ along the growth $k_{\left|\right|}$ ([001]), and in-plane $k_{⊥}$ ([100], [010]) directions. The SL phonon dispersions displayed flattening of modes, especially at high-symmetry critical points Γ, Z and M. Miniband formation and anti-crossings in $\omega_{j}^{SL}\left(\vec{k}\right)$ lead to the reduction in phonon conductivity $\kappa_{z}$ along the growth direction by an order of magnitude relative to the bulk materials. Due to zone-folding effects, the in-plane phonons in SLs exhibited a strong mixture of XC-like and YC-like low-energy $\omega_{TA}$, $\omega_{LA}$ modes with the emergence of stop bands at certain $\left|{\vec{k}}_{SL}\right|$. For thermal transport applications, the results demonstrate modifications in thermal conductivities via changes in group velocities, specific heat, and density of states.

## 1. Introduction

Elemental group-IV semiconductors, including diamond (C), silicon (Si), germanium (Ge), and tin (Sn), are the most technologically advanced electronic materials. Under ambient conditions, they crystallize in the diamond structure, except for α-Sn which is stable only at T < 290 K [1]. Notable advances have been made in comprehending the basic traits of group-IV materials due to their unique and exciting characteristics. The X_x_Y_1−x_ alloys (X, Y = C, Si, Ge, and Sn) and low-dimensional heterostructures (LDHs) (i.e., multi-quantum wells (MQWs) and superlattices (SLs)) are equally valuable for bandgap and strain engineering applications. The electronic industry has already employed Si_1−*x*_Ge_*x*_ [1] in many electronic devices as Si and Ge materials are miscible across the entire composition, x. These alloys have enabled the tuning of lattice constants $a_{o}$ and energy bandgaps $E_{g}$. In Si-based technology, it has been the aspiration of many scientists and engineers to develop light-emitting diodes (LEDs) and lasers by monolithically integrating them with complementary metal-oxide-semiconductors (CMOS). Despite the conceptual constraints of Si to generate light, the Si-centered optical platform has rapidly changed the landscape of photonic integrated circuits (PICs) by proposing robust solutions in the areas of telecom, datacom, bio-photonics and quantum networks [2,3,4,5,6,7,8,9,10,11,12,13,14], etc. Now, the concept of achieving direct bandgap with group IV carbides XC (X ≡ Si, Ge and Sn) has offered a paradigm shift in Si-photonics concerning the uniform implementation of light emitters. Thus, the growth of novel XC materials and their polymorphs on Si substrates has attracted considerable attention for advancing new opportunities [15,16,17,18] to create different device structures. The large lattice mismatch between XC epilayers and Si substrate with differences in thermal expansion coefficients trigger many structural and/or intrinsic defects near the film-substrate interfaces [18,19,20]. The appropriate choice of buffer layers acquiring load through relaxation of mechanical stresses in LDHs has helped improve the structural qualities of MQWs, and SLs [15,16,17,18,19]. There remain, however, a few intrinsic issues that could constrain the designing of some optoelectronic device structures. The solutions to these problems are not impossible and can be resolved by exploiting suitable experimental (e.g., growth, [18,19,20] characterization [21,22,23,24,25,26,27,28,29,30,31,32,33,34,35,36,37,38,39,40,41,42,43,44,45,46,47,48,49]) and/or theoretical methods [17,50,51,52,53,54,55,56,57,58,59,60,61]. Obviously, the group-IV carbides’ incredibly fascinating electronic and optical properties have made them exclusively unique from II-VI and III-V compound semiconductors [1] for further investigations.

Experimentally, there exist limited optical and structural studies of XC materials by exploiting infrared (IR), Raman scattering spectroscopy (RSS) [45], high-resolution X-ray diffraction (HR-XRD) [30,31,32,33,34], photoluminescence (PL) [34] and spectroscopic ellipsometry (SE) techniques [34], etc. Most efficient methods for assessing the complete phonon dispersions $\omega_{j}\left(\vec{k}\right)$ in group-IV and compound semiconductors have been found in the inelastic neutron scattering (INS) [48,49], and/or inelastic X-ray scattering (IXS) [46]. In polar bulk crystals, both IR and RSS measurements can offer information on the long wavelength optical phonons [45] (i.e., near |$\vec{k}$| → 0 in the Brillouin zone (BZ)). Except for 3C-SiC [45,46], no measurements of $\omega_{j}\left(\vec{k}\right)$ exist for zb GeC and SnC materials either by using IXS, RSS and/or IR methods. The INS technique cannot be employed to XC/Si [001] epifilms, because the epitaxially grown samples are too lean to obtain measurable signals to resolve the modes and branches of $\omega_{j}\left(\vec{k}\right)$ lying very close in frequency.

Theoretical efforts have also been made to study the phonon dispersions of bulk materials and SLs [17,50,51,52,53,54,55,56,57,58,59,60,61] using simple [52] and complex schemes [53,54,55,56,57,58,59,60,61,62,63,64,65,66,67,68,69,70,71,72,73,74,75,76,77,78,79,80,81]. For the bulk and layered structured materials, both elastic continuum and linear-chain models (LCMs) [52] are employed to study their limited portion of the vibrational spectrum (e.g., acoustic vibrations). Realistic lattice dynamical methodologies including rigid-ion-model (RIM) [50,51], full potential linear augmented plane wave (FP-LAPW), molecular dynamics (MD) and first-principles (ab-initio) approaches [53,54,55,56,57,58,59,60,61] can become challenging when dealing with complex structures and/or investigating phonon features of thick SLs. Earlier studies of phonon dispersions for bulk XC materials using FP-LAPW, ab initio and MD schemes provided results at variance [53,54,55,56,57,58,59,60,61] with each other. Recent RIM calculations [50,51] of $\omega_{j}\left(\vec{k}\right)$ for XC have agreed reasonably well with the results reported by Jankousky et al. [17] who used an ab initio random structure sampling method.

Except for LCM [52], there are no realistic calculations available for comprehending the phonon dispersions $\omega_{j}^{SL}\left(\vec{k}\right)$ of strained layer ${\left(XC\right)}_{m}/({YC)}_{n}$ SLs. Phonons are the primary carriers of sound and heat in solids, playing important roles in comprehending their acoustical, thermal, electronic, and optical properties [82,83,84,85,86,87]. In recent years, the tendency of miniaturized nanostructured electronic devices (NEDs) has been increasingly integrated and functionalized. Heat conduction thus becomes one of the most crucial research projects due to its prominence in many applications [88,89,90,91,92,93,94,95,96,97,98,99]. Despite the strong scientific and practical importance of C-based SLs, conclusive evidence of phonon scattering or confined acoustic/optical modes in individual and/or freestanding materials is lacking. If phonons are not scattered internally in layers or diffusively at interfaces, the zone folding can lead to many important effects changing the thermal transport in SLs.

Due to lower spatial symmetry, one would expect more phonon branches in SLs, triggering additional channels for phonon–phonon scattering [82,83,84,85,86,87]. New phonon bands in SLs can attain zero velocity at zone edges which experience an overall flattening to cause a reduction in phonon group velocities and thermal conductivities, κ [62,73]. Additionally, the interaction between phonons and other particles can pose resistance, especially when phonons interact with impurities and defects [88,89,90]. In a low temperature regime, the T-dependence κ exhibits an interesting transition from 3D Debye T^3^ law to an almost linear T behavior in nano-size materials [91,92]. Thus, understanding of κ in micro and nanostructures is of current scientific interest, especially for designing thermoelectric and/or cooling microelectronic devices. Novel C-based LDHs are materials whose vibrational modes differ markedly from their bulk counterparts. This could provide opportunities to scientists for tailoring their κ values by exploiting epitaxially growth techniques. Experimentally, an order of magnitude reduction in the component of lattice conductivity $\kappa_{z}$ was observed earlier along the growth direction [001] in GaAs/AlAs SLs [93] relative to the bulk GaAs. The κ value of Si/Ge SLs was also studied by Hyldgaard et al. [94], Chen et al. [95,96], and Tamura et al. [97]. Thus, it is imperative to investigate the possibility of reduction in κ associated with the changes in phonon dispersion $s\;\omega_{j}^{SL}\left(\vec{k}\right)\;$ of C-based SLs by using a realistic, computationally intensive lattice dynamical method [63].

This paper aims to report methodical simulations of the vibrational characteristics $\omega_{j}^{SL}\left(\vec{k}\right)$ on SLs by using a realistic lattice dynamical scheme. Unlike LCM, the effects of phonon folding are systematically investigated by using a RIM [63]. The method includes both the short- and long-range Coulomb interactions. Careful studies of $\omega_{j}^{SL}\left(\vec{k}\right)$ are accomplished in the growth [001] as well as in the in-plane [100], [010] directions by choosing different number of monolayers m, n, of the XC-YC constituents in ${\left(XC\right)}_{m}/({YC)}_{n}\;\left[001\right]$ SLs. In Section 2, we succinctly outlined the salient features of RIM [63] for constructing the dynamical matrix $D_{\alpha \beta }^{sC}$ of zb materials by including the general short-$D_{\alpha \beta }^{s},$ and long-range Coulomb $D_{\alpha \beta }^{C}$ interactions (cf. Section 2.1). For bulk XC materials, the effective electron transfer charge Ze (≡Z_eff_) and short-range forces up to 2nd next-nearest neighbors are accurately obtained by successive least-square fitting procedures [50]. In Section 2.2 and Section 2.3, we describe the appropriate modifications in the dynamical matrices of binary materials for studying the phonon dispersions $\omega_{j}^{SL}\left(\vec{k}\right)\;$, group velocities, density of states, and phonon conductivities of the SLs. Results of numerical simulations of $\omega_{j}^{SL}\left(\vec{k}\right)$ are reported in Section 3 for the phonon wavevectors parallel $k_{\left|\right|}$ (i.e., cross-plane) and perpendicular, $k_{⊥}$ (i.e., in-plane [100], [010], [110]) to the growth [001] direction. Comparison of the calculated phonon dispersions $\omega_{j}\left(\vec{k}\right)$ for the bulk XC materials provided reasonably good agreement with the experimental/ab-initio results (cf. Section 3.1 and Section 3.1.1 [A], [B], [C]). In Section 3.2 and Section 3.2.1, we outline the symmetry and selection rules for observing the IR and/or Raman active phonons in the zb materials, and their SLs. The impacts of bulk phonon dispersions $\omega_{j}\left(\vec{k}\right)$ on $\omega_{j}^{SL}\left(\vec{k}\right)$ of the SLs are briefly addressed (cf. Section 3.2.2 [A], [B], [C]). Comprehensive calculations of $\omega_{j}^{SL}\left(\vec{k}\right)$ reported in Section 3.2.3 [A], [B], [C] for different wavevectors ($|{\vec{k}}_{SL}|{\equiv k}_{\left|\right|}$, $k_{⊥}$) revealed the anisotropic phonon mode behavior, mixing of acoustic-phonons, anti-crossing with mini-gap formation, confinement of optical phonons (COPs) and their angular dependence, etc. Results of $\left|{\vec{k}}_{SL}\right|$ dependent modes for short-period SLs confirmed continuous frequency spectra in the mini-Brillouin zone (m-BZ). A comparison is made with the existing results of phonon characteristics in different SLs. Unlike the conventional (GaAs)_m_/(AlAs)_n_ structure, the RIM calculations of (SiC)_n_/(GeC)_n_ (or (SiC)_n_/(SnC)_n_) SLs revealed atypical phonon mode features. In particular, the SiC-like $\omega_{LA}$ modes fall in the phonon gap region which separates the optical bands of the bulk GeC-SiC (and/or SnC-SiC) materials. The theoretical results are compared/contrasted (cf. Section 3) with the existing data on several conventional semiconductor SLs. Phonon dispersions of SLs displayed the flattening of modes, especially at high critical points Γ, Z and M in the m-BZ. Miniband formation and anti-crossings in $\omega_{j}^{SL}\left(\vec{k}\right)$ lead to an order of magnitude reduction in $\kappa_{z}$ along the growth direction relative to the values of bulk materials. Conclusions are drawn in Section 4, reiterating the peculiar mode behavior in ${\left(XC\right)}_{m}/({YC)}_{n}$ SLs with general comments on how the confined acoustic phonons impacted their thermal properties.

## 2. Methodology

Several calculations of phonon dispersions on the conventional GaAs/AlAs, GaAs/InAs [001] SLs are reported in the literature by employing both simplified LCM [64,65], elastic, dielectric continuum models [66,67,68,69], as well as realistic Keating-type [70,71,72], adiabatic bond-charge [73], RIM [74], and valence overlap shell models [75,76]. Some of these studies have considered disordered interface layers. For phonon wavevector parallel $k_{\left|\right|}$ to the growth [001] direction of SLs, the LCM predicted accurate values of folded acoustic modes (FAMs) [52]. However, the model cannot be used to describe $\omega_{j}^{SL}\left(\vec{k}\right)$ with wavevector perpendicular $k_{⊥}$ to the growth [001] direction. Macroscopic dielectric continuum models [66,67,68,69] have explained the interface-, slab modes and anisotropy of optical phonons. Calculations of $\omega_{j}^{SL}\left(\vec{k}\right)\;$ are also performed using a 2-parameter Keating model [70,71,72] by including the long-range Coulomb interactions. Again, the Keating model [70,71,72] was unable to describe phonon dispersions and flatness of the transverse-acoustic branches in the bulk GaAs, and AlAs materials. Except for RIM [74], many of the above microscopic calculations did not address the questions about the anisotropy of zone-center optical phonons, interface modes, and/or the acoustic stopbands that have been observed experimentally [77,78] in the conventional GaAs/AlAs SLs.

Here, we adopted a realistic RIM (cf. Section 2.1) which has already provided accurate phonon dispersions and thermodynamic properties of the bulk XC materials [50]. In Section 2.2, the model is extended to ${\left(XC\right)}_{m}/({YC)}_{n}$ SLs for studying the FAMs, anisotropy of optical phonons, interface modes, and mixing of the low energy phonons for triggering the acoustic stopbands, etc. Calculations of $\omega_{j}^{SL}\left(\vec{k}\right)$ are reported for the ${\left(XC\right)}_{m}/({YC)}_{n}$ SLs (cf. Section 3) by selecting different numbers of monolayers, m, n and phonon wavevectors both $k_{\left|\right|}$ and $k_{⊥}$ to the growth [001] direction, exhibiting a strong involvement of low and high-frequency phonon modes. Anisotropic thermal conductivities in ${\left(XC\right)}_{n}/({YC)}_{n}$ are also computed as a function of n (cf. Section 2.3). We must emphasize that this work is primarily concerned with simulating the effects of SL-induced changes in phonon dispersion $\omega_{j}^{SL}\left(\vec{k}\right)$ on the lattice thermal conductivity, κ. Since the SLs are regarded as perfect manmade periodic lattices (with no impurities), the ideal structures under discussion automatically include the coherent effects associated with the perfect interfaces.

### 2.1. Lattice Dynamics of Bulk XC Materials

Phonon dispersions of bulk zb materials with translational symmetry can be obtained by using a linear response approach. In RIM, the force $F_{\alpha }\left(l,\kappa \right)$ on κ^th^ atom in the l^th^ unit cell of bulk XC crystal can be written as [63]:

(1) $$F_{\alpha }\left(l,\kappa \right)=\sum_{l^{\prime }\kappa^{\prime }\beta }\Phi_{\alpha \beta }\left(l,\kappa ,l^{\prime }\kappa^{\prime }\right)u_{\beta }\left(l^{\prime }\kappa^{\prime }\right),$$

where α and β are the Cartesian coordinates; $u_{\alpha }$ is the displacement in the α (=x, y, and z) direction and the term $\Phi_{\alpha \beta }\left(l,\kappa ,l^{\prime }\kappa^{\prime }\right)$ represents the atomic force constants. In the harmonic approximation, and assuming a traveling wave solution, the atomic displacement $u_{\alpha }$ of the j^th^ mode $\omega_{j}(\vec{k})$ with wave-vector $\vec{k}$ can be expressed as:

(2) $$u_{\alpha }\left(l\kappa |\vec{k}j\right)=\frac{1}{\sqrt{M_{\kappa }}}e_{\alpha }(\kappa |\vec{k}j)e^{i[\vec{k}.\vec{x}(l\kappa )-\omega_{j}(\vec{k})t]},$$

In Equation (2), the term t identifies time; $\vec{x}(l\kappa )$ and $M_{\kappa }$, represent, respectively, the position and mass of the (lκ) atom. In RIM, it is straightforward to obtain the eigenvalue equations of motion [63]:

(3) $$\omega_{j}^{2}\left(\vec{k}\right)e_{\alpha }\left(\kappa |\vec{k}j\right)=\sum_{\kappa \prime \beta }D_{\alpha \beta }^{sC}(\kappa \kappa \prime |\vec{q})e_{\beta }(\kappa \prime |\vec{k}j);\;\kappa ,\kappa^{\prime }=1,2$$

where, $D_{\alpha \beta }^{sC}(\kappa \kappa \prime |\vec{k})$ [≡$D_{\alpha \beta }^{s}(\kappa \kappa \prime |\vec{k})$ +$\;D_{\alpha \beta }^{C}(\kappa \kappa \prime |\vec{k})$] represents the dynamical matrix comprising of short-$D_{\alpha \beta }^{s}(\kappa \kappa \prime |\vec{k})$, and long-range Coulomb $D_{\alpha \beta }^{C}(\kappa \kappa \prime |\vec{k})$ interactions. In defining the short-range part $D_{\alpha \beta }^{s}(\kappa \kappa \prime |\vec{k})$ of the bulk XC materials, there are two nearest-neighbor A, B and eight next-nearest-neighbor C_1_, D_1_, E_1_, F_1_, C_2_, D_2_, E_2_, and F_2_ interatomic force constants (IFCs) [63] which describe the interactions between anions (κ= 1) and cations (κ= 2), respectively. In RIM, the strength of electrostatic interaction in the long-range Coulomb part of the dynamical matrix $D_{\alpha \beta }^{C}(\kappa \kappa \prime |\vec{k})$ is carefully included in terms of the effective ionic charge Z_eff_ [50]. All the IFCs are accurately assessed [50] for the zb XC materials by instigating the least-square fitting procedures to achieve a very good fit to the experimental [45,46] and/or ab initio results [17,53,54,55,56,57,58,59,60,61] of the phonon frequencies at high critical points in the BZ.

### 2.2. Lattice Dynamics of XC/YC Superlattices

By involving the phonon dispersions $\omega_{j}(\vec{k})$ of bulk XC, YC materials, the extension of RIM for simulating $\omega_{j}^{SL}\left(\vec{k}\right)$ in (XC)_m_/(YC)_n_ (001) SLs is straightforward. Systematic construction of the short-range dynamical matrix of SL can be achieved in terms of the (6 × 6) size dynamical matrix of the bulk constituents XC and YC. To accomplish this, we defined an atomic layer as a collection of atoms in a plane normal to the growth direction, a bilayer as two adjacent atomic layers, and a sublattice as the collection of all the equivalent atomic layers (one in each period) of the superlattice. In (XC)_m_/(YC)_n_, each period of the SL is arranged by using m bilayers of the XC and n bilayers of the YC constituent materials. In this setting, the entire superlattice system is composed of 2 (m + n) sublattices. Following Ren et al. [74], we considered a label χ for identifying the bilayers in each period and (χ, s) for the two atomic layers associated with the bilayer χ, where s = 1 is implied for the cation layers and s = 2 for the anion layers. Similarly, the sublattices are classified by using (χ, s).

For constructing the dynamical matrix of (XC)_m_/(YC)_n_ SLs, we retained the short-range parameters of the bulk constituents XC and YC and meticulously incorporated the long-range Coulomb interactions. In our adaptation of the RIM, the short-range IFCs between any two atoms in the SL are kept the same as in the bulk materials; however, the interaction between the X and Y atoms across the SL interface is taken as an average of the X-X and Y-Y IFCs of the bulk constituents. Due to large splitting in the optical phonons ${∆\omega }^{opt}\;[(\equiv \omega_{LO\left(\Gamma \right)}$ − $\omega_{TO(\Gamma )})]$ at the center of the BZ for XC and YC materials, the long-range Coulomb interactions in SLs cannot be ignored for simulating $\omega_{j}^{SL}\left(\vec{k}\right)$.

A basic theory of how the long-range Coulomb interactions can be considered in the RIM, has been described in several books and monographs. These include the excellent work by Born and Huang [79], Maradudin et al. [80], and Venkataraman et al. [81], etc. The connection between macroscopic and microscopic theories of bulk crystals has also been established. However, in SLs it is more difficult to handle the long-range Coulomb interaction due to its lower symmetry. One must note that the Ewald transformation which ensures the fast convergence for evaluating the Coulomb interaction in bulk crystals must be modified for the SLs. In RIM and following Ren et al. [74], we obtained the Coulomb interactions in short-period SLs by considering the Madelung sum and its derivatives using a generalized Ewald transformation procedure with enlarged unit cells. This approach between any two layers of the ions is used for computing the Coulomb matrix elements with fast convergence. The resulting interactions between sublattice (0,0) and another at (χ, s) in the long-wavelength limit (i.e., $\left|\vec{k}\right|$→ 0) can be expressed as [74]:

(4) $$C_{ij}^{C}\left(\chi ,r_{s}|\vec{k}\right)=\left(\frac{{Z_{0}Z}_{s}}{Ω}\right)\frac{4\pi }{N}\left(\frac{k_{i}k_{j}}{\left|{\vec{k}}^{2}\right|}\right)+C_{ij}^{sr}(\chi ,r_{s}),$$

where i, j indicates the x, y, z, directions, $Z_{0}$ and $Z_{s}$ represent the atomic transfer charges of atoms in sublattice (0, 0) and (χ, s), Ω is the volume of the bulk unit cell, and N = (n + m) is the total number of bilayers in the enlarged SL period. The term $C_{ij}^{sr}$ is the short-range part of the Coulomb matrix, which may be truncated at a short distance without losing too much accuracy. The bulk XC electron transfer charge $Z_{a}$ is used for the inner XC layers, and the bulk YC electron transfer charge $Z_{b}$ for inner YC layers. At the interfaces, the transfer charge of C is taken to be the average of $Z_{a}$ and $Z_{b}$ since the electron overlapping is significant only to the nearest neighbors. Details of the numerical method used for calculating $C_{ij}^{C}\left(\chi ,r_{s}|\vec{k}\right)$ for (XC)_m_/(YC)_n_ SLs can be found in Ref. [74]. The IFCs for bulk materials used to simulate $\omega_{j}^{SL}\left(\vec{k}\right)$ are taken from our recent publication [50].

### 2.3. Thermal Conduction in Superlattices

Lattice thermal conductivity (κ) can strongly affect the applications of many LDHs allied to thermal functionality, including thermal management, thermal barrier coating and thermoelectric, etc. In bulk semiconductors, κ is simulated in terms of specific heat, phonon group velocity, and phonon relaxation time by linking them to their phonon dispersions. Different materials of low and/or high κ are achieved with careful manipulations of these parameters. In the constant mode relaxation time approximation, κ in SLs can be obtained by summing the contributions of phonon modes $\omega_{j}^{SL}\left(\vec{k}\right)$ by using [62]:

(5) $$\kappa_{i}=\sum_{j}C_{ph}\left(\omega_{j}^{SL}\right){\left(v_{gi}\right)}^{2}\tau ,$$

where $C_{ph}$ is the mode-specific heat related to SL vibrational frequencies $\omega_{j}^{SL};$ i identifies the direction of thermal conduction; $v_{gi}\;\;is\;the$ group velocity (with components in the in-plane $v_{gx}$ and growth $v_{gz}\;$ directions); the term τ is the phonon relaxation time. Phonon group velocities determined from the vibrational spectrum can be expressed as [62]:

(6) $$v_{gj}\left(\vec{k}\right)=\frac{\partial \omega_{j}^{SL}\left(\vec{k}\right)}{\partial k}.$$

The model that we adopted here for simulating the normalized phonon thermal conductivities $\kappa^{\prime }\left(\equiv \frac{\kappa_{SL}}{\kappa_{avg}};\kappa_{z}^{\prime },\;\kappa_{x}^{\prime }\right)$ of (XC)_n_/(YC)_n_ SLs is successfully applied in several investigations to comprehend the lattice dynamical properties of perfect/imperfect semiconductors. Phonon dispersions $\omega_{j}^{SL}\left(\vec{k}\right)$ of SLs with wavevectors parallel $k_{\left|\right|}\;and$ perpendicular $k_{⊥}$ to the growth directions are used in Equations (5) and (6) to calculate the normalized thermal conductivities $\kappa_{z}^{\prime }$ $and\;\kappa_{x}^{\prime }$ as a function of n. A challenge is encountered, especially near the sites where phonon branches are crossing to keep track of each mode.

## 3. Numerical Computations, Results and Discussions

**Figure 1:**
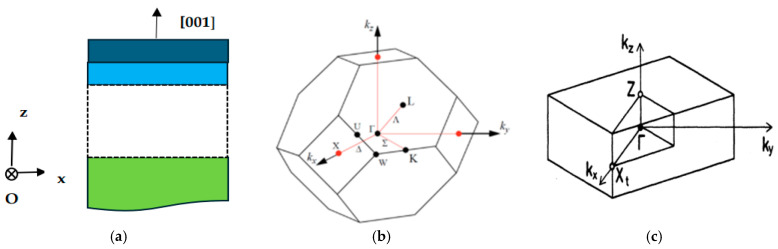
(**a**) Superlattice structure using XC (dark blue) and YC (sky blue color) materials stacked alternately along the [001] or z-direction to create (XC)_m_/(YC)_n_ superlattice on a substrate (green color). (**b**) The Brillouin zone of face-centered cubic material labelling different high-symmetry critical points (Γ, X, K, L W, etc.). (**c**) The Brillouin zone of a (XC)_m_/(YC)_n_ superlattice with m + n an even integer, the point X_t_, in (**c**) is the same as the X point in (**b**) (see text).

Lattice dynamics $\omega_{j}\left(\vec{k}\right)$ of bulk semiconductors relate the phonon mode frequencies to its discrete wave-vector $\vec{k}$. An artificial structure of ${\left(XC\right)}_{m}/({YC)}_{n}\;SL$ can be prepared by stacking alternate layers of two different bulk XC (dark blue), and YC (sky blue) constituent materials in the z-direction (Figure 1a) of thicknesses d_1_ and d_2_, respectively.

The largest allowed $\left|\vec{k}\right|$ value in the bulk zb material is related (Figure 1b) to inverse of its lattice constant $a_{0}$. As the period of SL is increased to d_SL_ composed of 2 (m + n) sublattices in the growth [001] z-direction, one expects reduction in phonon wavevector $|\vec{k}|(\equiv$2π/$a_{0})$ from bulk BZ (Figure 1b) to $\left|{\vec{k}}_{SL}\right|$ (≡π/d_SL_) in the m-BZ. This reduction in $\left|{\vec{k}}_{SL}\right|$ (Figure 1c) prompts folding of the bulk phonon modes [74] in SLs.

### 3.1. General Characteristics of Phonons in Bulk Materials

Recent studies on the lattice dynamics of novel XC materials have provided information for their dynamic stability [17]. By exploiting RIM [63] and using optimization methods with the non-linear least square fitting procedures, we carefully evaluated the necessary IFCs for the bulk zb SiC, GeC and SnC materials to comprehend their vibrational, thermodynamic and structural, characteristics [50]. Before presenting the results of the vibrational properties for (SiC)_m_/(GeC)_n_, (SiC)_m_/(SnC)_n_, and (GeC)_m_/(SnC)_n_ SLs (cf. Section 3.1.1), we reported results of phonon dispersions and one phonon density of states (DOS) for the SiC-GeC, SiC-SnC and GeC-SnC binary materials, involved in the constructions of appropriate SLs. Good comparison/contrast of RIM results $\omega_{j}\left(\vec{k}\right)$ for the bulk materials with existing experimental/theoretical data (cf. Section 3.1.1) gave us the confidence to extend this approach to evaluate phonon dispersions $\omega_{j}^{SL}\left(\vec{k}\right)\;$ of ${\left(XC\right)}_{m}/({YC)}_{n}$ SLs.

#### 3.1.1. Lattice Dynamics of Bulk Materials

By using the RIM, we calculated phonon dispersions of the bulk compounds $\omega_{j}\left(\vec{k}\right).$ One-phonon density of states (DOS) $g\left(\omega \right)$ for XC materials are obtained by carefully incorporating the phonon frequencies at a mesh of 64,000 $\left|\vec{k}\right|$ point in the BZ. Standard practices are adopted for numerical calculations of $g\left(\omega \right)$ by setting the phonon sampling widths Δω (≡$\omega_{LO(\Gamma )}/$100). With respect to the common C anion in XC materials, the results of $\omega_{j}\left(\vec{k}\right)$ and $g\left(\omega \right)$ are seen significantly changing with the variation of masses of the cations: i.e., from Si (28.1 amu) → Ge (72.6 amu) → Sn (118.7 amu). Theoretical results are compared/contrasted against the existing experimental [45,46] and first-principles [17,53,54,55,56,57,58,59,60] calculations. Our results revealing distinct features of $\omega_{j}\left(\vec{k}\right)$ and $g\left(\omega \right)$ in bulk SiC-GeC, SiC-SnC, and GeC-SnC binaries (cf. subsections [A], [B], and [C]) are expected to impact on the phonon dispersions $\omega_{j}^{SL}\left(\vec{k}\right)$ of their ${\left(XC\right)}_{m}/({YC)}_{n}$ SLs.

##### [A] SiC-GeC 

**Figure 2:**
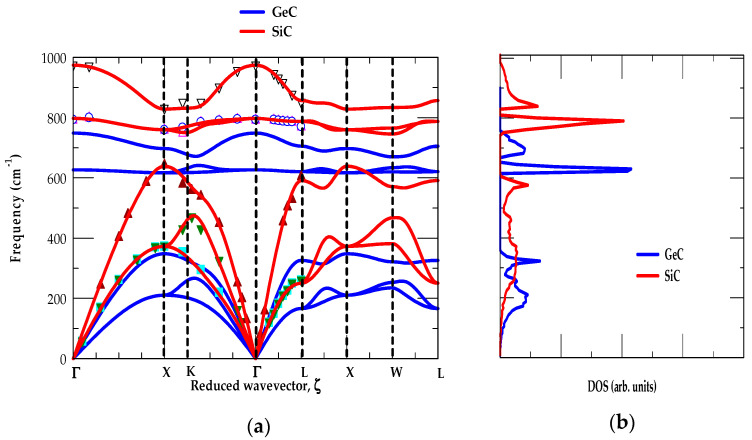
(**a**) Comparison of the rigid-ion-model (RIM) calculations of 3C-SiC (red colored lines) with the experimental (inelastic X-ray scattering, IXS using symbols) and zb GeC (blue colored lines) along high-symmetry directions of the BZ Brillouin zone, (**b**) RIM calculated results of one-phonon density of states $g\left(\omega \right)$ for 3C-SiC (red colored lines) and zb GeC (blue colored lines) (see text).

Figure 2a,b, shows RIM results of $\omega_{j}\left(\vec{k}\right)$ and $g\left(\omega \right)$ for the zb SiC and GeC materials, respectively. The $\omega_{j}\left(\vec{k}\right)$ along high-symmetry directions (Γ → X → K → Γ → L → X → W → L) and $g\left(\omega \right)$ are displayed using red-, and blue-colored lines. Theoretical results of bulk materials are compared/contrasted reasonably well with the available experimental (RSS and IXS) [45,46] and simulated first-principles data of Zhang et al. [53].

Figure 2a shows three interesting phonon characteristics of the SiC-GeC materials: (i) the low-frequency acoustic modes are initiated by the heavier Si (28.1 amu) and Ge (72.6 amu) atomic vibrations, (ii) the high-frequency optical modes are instigated by the light C (12.0 amu) atomic vibrations, and (iii) the acoustic ($\omega_{TA}$) phonon branches in SiC-GeC exhibited positive values, indicating their stability in the zb structure at ambient conditions [17]. In SiC (GeC), the major acoustical ($\omega_{LA},\omega_{TA1}$, $\omega_{TA2}$) modes fall below its phonon band gap region between the 618–748 cm^−1^ (358–620 cm^−1^) frequency. Clearly, this caused a slight overlap of SiC $\omega_{LA}$ mode (red-colored line) on the lower portion of the GeC optical phonon (blue-colored line). Again, their g(ω) (see Figure 2b) reveals two low energy features (below the phonon gap), attributing to the average values of $\omega_{TA}$ and $\omega_{LA}$ modes instigated by the heavier Si (Ge) atomic vibrations. The other two high-frequency traits (above the phonon gap) in $g\left(\omega \right)$ are ascribed to the average values of $\omega_{TO}$ and $\omega_{LO}$ phonons due to the light C atomic vibrations in good agreement with the ab initio [17,53,54,55,56,57,58,59,60] calculations.

Moreover, the calculations of $\omega_{j}\left(\vec{k}\right)$ for zb SiC and GeC displayed nearly flat dispersions of $\omega_{TO}$ modes along the X → Γ → L directions (see Figure 2a). Consequently, these results are responsible for inducing strong optical phonon peaks in the one-phonon (cf. Figure 2b) DOS g(ω). On the other hand, our RIM studies of $\omega_{j}\left(\vec{k}\right)$ have shown $\omega_{LO}$ mode dispersions becoming nearly flat along the L → X → W directions. These results are related to initiating relatively weak peaks in their respective one-phonon DOS g(ω).

##### [B] SiC-SnC

**Figure 3:**
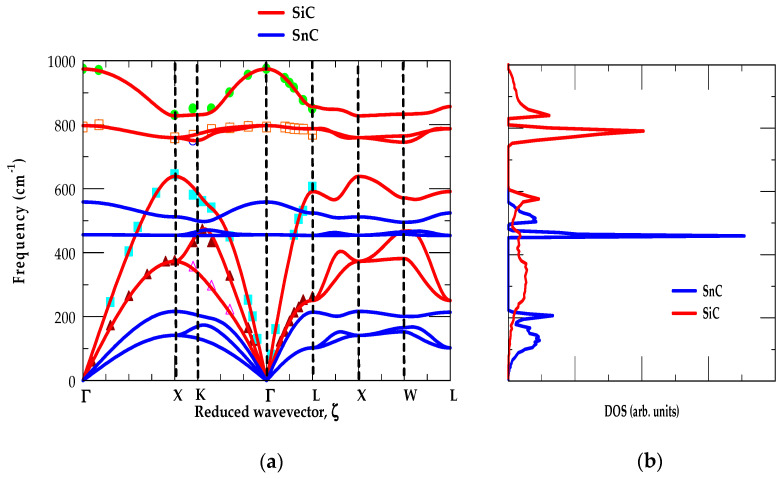
(**a**) Comparison of the rigid-ion-model (RIM) calculations of 3C-SiC (red color lines) with the experimental (inelastic X-ray scattering, IXS using symbols) and zb GeC (blue color lines) along high-symmetry directions of the BZ Brillouin zone, (**b**) RIM calculated results of one-phonon density of states $g\left(\omega \right)$ for 3C-SiC (red color lines) and zb GeC (blue color lines).

In Figure 3a,b, we displayed our results of phonon dispersions and DOS for zb SiC and SnC materials, respectively. The results of $\omega_{j}\left(\vec{k}\right)$ along high-symmetry directions (Γ → X → K → Γ → L → X → W → L) (see left panel) and their $g\left(\omega \right)$ (right panel) are indicated using red-, and blue-colored lines. Theoretical calculations of SiC and SnC materials are compared reasonably well with the available experimental (RSS and IXS) [45,46] as well as the first-principles data of Zhang et al. [53].

Like SiC-GeC, the examination of Figure 3a,b shows three interesting traits: (i) the low-frequency modes are initiated by heavier (Si (28 amu) and Sn (118.7 amu)) atomic vibrations, (ii) the high-frequency modes are caused by light (C (12 amu)) atomic vibrations, and (iii) the acoustic phonon branches in SiC- SnC exhibit positive values, indicating their stability in zb structure at ambient conditions. In SiC (SnC), the major acoustical $\omega_{LA},\omega_{TA1}$, $\omega_{TA2}$ modes fall below the phonon band gap between frequency 618–748 cm^−1^ (220–457 cm^−1^) region. Unlike SiC-GeC, (see Figure 3a,b) the $\omega_{j}\left(\vec{k}\right)$ results triggered a large portion of SiC $\omega_{LA}$ modes (red-colored line) overlapping and crossing the SnC optical phonons (blue-colored line) due to large differences in masses of the Si and Sn atoms.

Figure 3b reveals two low energy features, below the phonon gap, attributed to the average values of $\omega_{TA}$ and $\omega_{LA}$ modes caused by heavier Si (Sn) atomic vibrations. The other two high-frequency traits, above the phonon gap, in $g\left(\omega \right)$ are ascribed to the average values of $\omega_{TO}$ and $\omega_{LO}$ due to light C atomic vibrations corroborating the ab initio calculations [53]. Like SiC-GeC, the calculations of $\omega_{j}\left(\vec{k}\right)$ for SiC-SnC materials exhibited nearly flat dispersions of $\omega_{TO}$ modes along the X → Γ → L directions (see Figure 3a) for inducing strong optical phonon peaks in (cf. Figure 3b) g(ω). However, the $\omega_{LO}$ mode dispersions in $\omega_{j}\left(\vec{k}\right)$ are nearly flat along the L → X → W directions which produced relatively weak peaks in their respective one-phonon DOS g(ω).

##### [C] GeC-SnC

**Figure 4:**
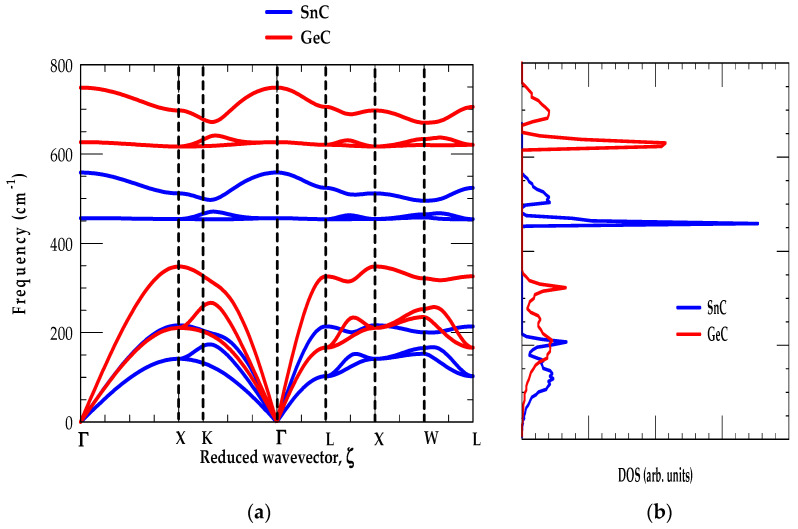
(**a**) Comparison of the rigid-ion-model (RIM) calculations of 3C-SiC (red color lines) with the experimental (inelastic X-ray scattering, IXS using symbols) and zb GeC (blue color lines) along high-symmetry directions of the BZ Brillouin zone, (**b**) RIM calculated results of one-phonon density of states $g\left(\omega \right)$ for 3C-SiC (red color lines) and zb GeC (blue color lines).

In Figure 4a,b, we displayed our simulated results of phonon dispersions and DOS for GeC and SnC materials, respectively. The $\omega_{j}\left(\vec{k}\right)$ along high-symmetry directions (Γ → X → K → Γ → L → X → W → L) and their $g\left(\omega \right)$ are shown using red-, and blue-colored lines. The RIM results for zb GeC and SnC materials are compared/contrasted reasonably well with the first-principles calculations of Zhang et al. [53].

The examinations of Figure 4a,b show traits similar to the conventional strained layer GaN/InN [76] SLs: (i) the low-frequency modes in GeC-SnC are initiated by heavier (Ge (72.6 amu) and Sn (118.7 amu)) atomic vibrations, (ii) the high-frequency modes are caused by the light (C (12.0 amu)) atomic vibrations, and (iii) the acoustic phonon branches exhibit positive values, indicating their stability in the zb structure at ambient conditions. In GeC (SnC), the major acoustical $\omega_{LA},\omega_{TA1}$, $\omega_{TA2}$ modes fall below its phonon band gap between the frequency 358–620 cm^−1^ (220–457 cm^−1^) region.

Obviously, these results (see Figure 4a,b) initiated a large separation of GeC optical modes (red-colored line) from those of the SnC optical phonons (blue-colored line). Again, Figure 4b reveals two low energy features (below the phonon gap), attributed to the average values of $\omega_{TA}$ and $\omega_{LA}$ modes caused by heavier Ge (Sn) atomic vibrations. The other two high-frequency features (above the phonon gap) in $g\left(\omega \right)$ are ascribed to the average values of $\omega_{TO}$ and $\omega_{LO}$ phonons due to the light C atomic vibrations corroborating the ab initio calculations. 

Like zb SiC-GeC and SiC-SnC, systems our studies of $\omega_{j}\left(\vec{k}\right)$ for GeC-SnC exhibited nearly flat dispersions of $\omega_{TO}$ modes along the X → Γ → L directions (see Figure 4a) for inducing strong optical phonon peaks in the one-phonon (cf. Figure 4b) DOS. On the other hand, the $\omega_{j}\left(\vec{k}\right)$ revealed $\omega_{LO}$ mode dispersions becoming nearly flat along the L → X → W directions for triggering relatively weak peaks in their respective one-phonon DOS g(ω). 

In Table 1, we reported RIM phonon frequencies at high critical points (Γ, X, L) in the BZ and splitting of optical modes ${∆\omega }^{opt}$ (≡$\omega_{LO\left(\Gamma \right)}{-\omega }_{TO(\Gamma )}$) in the zb SiC, GeC and SnC. The results are compared with the existing [45,46] experimental and/or theoretical [17,53,54,55,56,57,58] data. Table 1 clearly reveals that for the zb SiC, GeC and SnC materials, the discrepancies between RIM phonon frequencies at Γ, X, and L critical points and experimental (Raman, IXC [45,46]) data as well as the ab initio calculations [17,53,54,55,56,57,58] are less than 3%.

### 3.2. Phonon Characteristics in Superlattices

In the long wavelength limit, the measurements of Raman scattering and/or IR spectroscopies offer valuable information on phonons in polar materials and their SLs. Theoretically, the diagonalization of dynamical matrices for the zb XC-YC binary materials and their ${\left(XC\right)}_{m}/({YC)}_{n}$ SLs can be achieved at several wavevectors for comprehending their phonon characteristics. For different types of modes, the point-group symmetries helped obtain additional information about their crystalline structures. Before presenting comprehensive RIM results of phonon dispersions $\omega_{j}^{SL}\left(\vec{k}\right)$ for the SLs (cf. Section 3.2.2 [A]–[C] and Section 3.2.3 [A]–[C]), we briefly outlined the selection rules using Group theoretical arguments (see Section 3.2.1) for detecting different types of phonon modes in zb polar materials and their SLs.

#### 3.2.1. Symmetry and Selection Rules

Polar zb XC and YC materials are face-centered-cubic with a tetrahedral $T_{d}$ point group symmetry with two atoms per primitive unit cell. In the long wavelength limit, (i.e., at zone-center $\left|\vec{k}\right|$ = 0, of bulk BZ), the optical phonons are triply degenerate with symmetry species $\Gamma_{15}$(F_2_). By first-order Raman scattering, the doubly degenerate $\omega_{TO(\Gamma )}$ and a non-degenerate $\omega_{LO(\Gamma )}$ mode of higher frequencies are observed, and their identity is established by using polarization characteristics in the IR and RSS studies [100,101].

A periodic ${\left(XC\right)}_{m}/({YC)}_{n}$ SL is grown in the z-direction $\left[001\right]$ using two XC, YC constituents by considering m = n number of monolayers. This artificial structure induces a reduction in symmetry from the $T_{d}$ to the tetragonal of the point group $D_{2d}.$ The symmetry considerations can provide guidance on different forms of Raman tensors for the uniaxial materials. Group theory, however, determines their exact types in the various crystal orientations. In ${\left(XC\right)}_{n}/({YC)}_{n}$[001] SLs, there are 4 n atoms involved in a larger primitive unit cell with a total of 12 n vibrational modes for each superlattice wave-vector $\left|{\vec{k}}_{SL}\right|$. By using Group theoretic arguments, the phonons of $D_{2d}$ symmetry are distributed in the following irreducible representations [100,101]:

(7) $$\Gamma_{D_{2d}}\;=2\left(B_{2}+E\right)+\left(2n-1\right)\left(A_{1}+B_{2}+2E\right),$$

where, $A_{1}$ type of mode is Raman active, while the transverse E (doubly degenerate), and $B_{2}\;type$ (longitudinal) of modes are both Raman and IR active. At |${\vec{k}}_{SL}$| = 0 of the SL m-BZ, the displacement field (strain) produced phonons of $A_{1}$ type is symmetric and phonons of $B_{2}$ types antisymmetric with respect to the center of each layer. Their corresponding Raman tensors are expressed in [100,101].

In the $A_{1}$ representation, only the diagonal elements of the Raman tensor are non-zero. This means that one can observe $A_{1}$ modes which couple to light by photo elastic mechanism in the $z(xx)\bar{z}$ geometry, where the polarization of incident and the scattered radiations are both in the x-direction; z and $\bar{z}$ signifies the incoming and outgoing directions. The bar at the top of z means an opposite direction of z. It is associated with the fact that the polarization of incident and scattered radiations is both in the x-direction. In the $B_{2}$ representation, however, the polarization of the incident and scattered light is either in the x and y [$z\left(xy\right)\bar{z}]$ or y and x $\left[z\left(yx\right)\bar{z}\right]$ directions. At finite |${\vec{k}}_{SL}$|, as in the case of backscattering geometry of Raman experiments, both $A_{1}$ and $B_{2}$ modes contain some admixture of |${\vec{k}}_{SL}$|~0 $A_{1}$ mode, giving rise to doublets. In general, $B_{2}$ type modes are observed in the first-order Raman scattering while the $A_{1}$ type of modes is not seen in the same order scattering due to small diagonal Raman tensor components [101]. This has instigated the development of the resonance RSS technique with several interesting features and conditions.

For the group of finite wavevectors $\left|{\vec{k}}_{SL}\right|$ in layers parallel to the SL axis, the point group symmetry becomes $C_{2v}$(orthorhombic) with $A_{1}$ type of symmetric longitudinal-, and $B_{1}$, $B_{2}\;types$ as the transverse modes. For wavevectors perpendicular (i.e., Γ → M direction) to the SL axis and for small ${\vec{k}}_{SL}\;$(i.e., |${\vec{k}}_{SL}$| → 0) forming an angle θ with *z*O*x* plane (see Figure 1a), the symmetry is $C_{s}$ of a monoclinic system. Thus, the zone center $B_{2}$ modes transform to $A^{\prime }$ while E modes split into $A^{\prime }$ and $A^{\prime \prime },$ respectively. The $A^{\prime }$ modes are mixed modes with the atomic displacement components along the *x* and *z* directions while the $A^{\prime \prime }$ modes are transverse in nature with ions vibrating along the Oy direction with constant frequency (dispersion less)—independent of angle θ. This is because these modes do not create the macroscopic field, while the $A^{\prime }$ modes exhibit a mixed polarization showing dispersion with θ due to the macroscopic field associated with them.

Again, the *z*-like $\omega_{{LO}_{n}}$ (n = odd) modes are classified as $B_{2}$ and (n = even) as $A_{1}$. The doubly degenerate x- and y-like $\omega_{{TO}_{n}}$ phonons are categorized as E modes [100,101]. The symbols $\omega_{{LO}_{n}}$ and $\omega_{{TO}_{n}}$ are designated to the nth quantized modes derived from the bulk (XC or YC) LO and TO phonons, respectively. No mixing occurs between the modes of different n. Again, for $A_{1}$($B_{2}$) modes, the atomic displacements oscillate symmetrically (anti-symmetrically) about the midplane in XC or YC layers [100,101].

#### 3.2.2. Phonons in Superlattices

**Figure 5:**
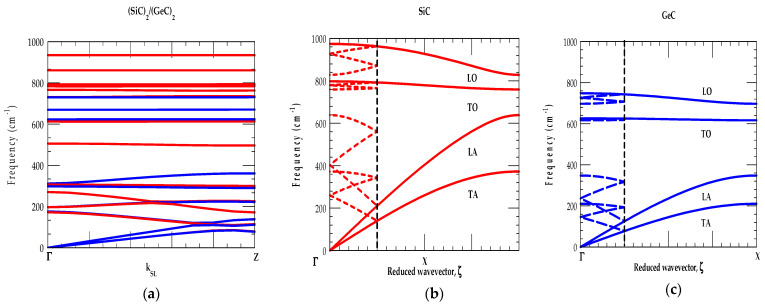
(**a**) Rigid-ion-model calculations of (SiC)_2_/(GeC)_2_ [001] SL in the growth (Γ → Z) direction. and zb GeC (blue color lines) along high-symmetry directions of the BZ Brillouin zone, (**b**) SiC bulk phonon dispersion curves along [001] (Γ → X) (red-color line) folded over the same period (dotted-line) as (**a**,**c**) GeC bulk phonon dispersion curves along [001] (Γ → X) (blue-color line) folded over the same period (dotted-line) as (**a**).

**Figure 6:**
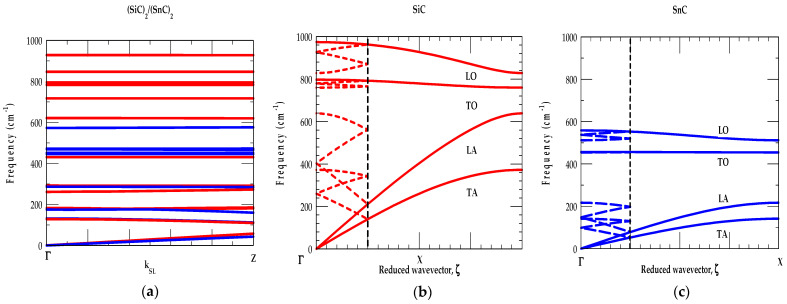
(**a**) Rigid-ion-model calculations of (SiC)_2_/(SnC)_2_ [001] SL in the growth (Γ → Z) direction. and zb GeC (blue color lines) along high-symmetry directions of the BZ Brillouin zone, (**b**) SiC bulk phonon dispersion curves along [001] (Γ → X) (red-color line) folded over the same period (dotted-line) as (**a**,**c**) SnC bulk phonon dispersion curves along [001] (Γ → X) (blue-color line) folded over the same period (dotted-line) as (**a**).

**Figure 7:**
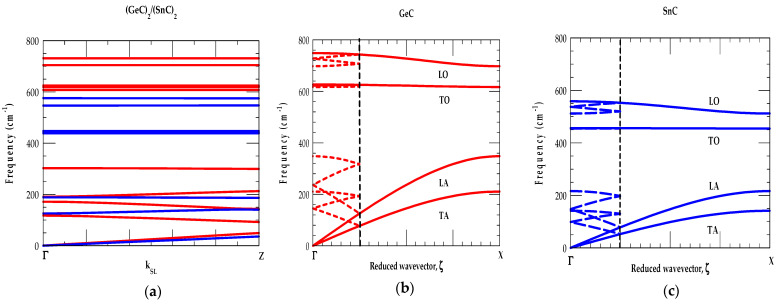
(**a**) Rigid-ion-model calculations of (GeC)_2_/(SnC)_2_ [001] SL in the growth (Γ → Z) direction. and zb GeC (blue color lines) along high-symmetry directions of the BZ Brillouin zone, (**b**) GeC bulk phonon dispersion curves along [001] (Γ → X) (red-color line) folded over the same period (dotted-line) as (**a**,**c**) SnC bulk phonon dispersion curves along [001] (Γ → X) (blue-color line) folded over the same period (dotted-line) as (**a**).

To simulate the phonon dispersions $\omega_{j}^{SL}\left(\vec{k}\right)$ of ${\left(XC\right)}_{n}/({YC)}_{n}$ SLs using RIM, we developed both the equations of motion and dynamical matrices by considering ten short-range interactions [74], and Coulomb interactions between all atoms within the SL. The later interactions are evaluated using Ewald’s summation technique. In Figure 5a–c, Figure 6a–c and Figure 7a–c we reported results of calculated phonon dispersion $\omega_{j}^{SL}\left(\vec{k}\right)$ for the (XC)_2_/(YC)_2_ [001] SLs, as well as phonon dispersions $\omega_{j}\left(\vec{k}\right)$ of the bulk XC and YC materials in the Γ → X directions. The folding of phonons over the same period as of the SLs m-BZ (see Figure 1c) is also shown. In sections [A], [B] and [C], we compared/contrasted and discussed the individual cases of three different C-based superlattice systems.

##### [A] (SiC)_n_/(GeC)_n_

The phonon dispersions $\omega_{j}^{SL}\left(\vec{k}\right)$ of (SiC)_2_/(GeC)_2_ SL are displayed in Figure 5a along the [001] (Γ → Z) direction. For comparison, the dispersion $\omega_{j}\left(\vec{k}\right)$ along the (Γ → X) direction of the bulk SiC (red-colored lines) (Figure 5b) and GeC (Figure 5c) (blue-colored lines) materials are reported. Using dotted red- and blue-colored lines, the folding of modes is also shown in the m-BZ of the SL (i.e., ¼ of the bulk Brillouin zone). Except for a small overlap of the $\omega_{LA}$ SiC mode on GeC optical phonons, the high-frequency optical modes $(\omega_{LO}$−$\omega_{TO}$) are well separated. This means that SL modes derived from the bulk optical phonon branches are either GeC-like (confined to GeC layers between (749–626 cm^−1^)) or SiC-like (confined to SiC layers between (974–793 cm^−1^)).

The degree of confinement can be determined by the smallest-imaginary-$\vec{k}$ solution to the corresponding bulk dynamical equation at a given frequency [74]. Earlier calculations of the decay lengths for the optical modes in SLs revealed that it is less than two atomic layers. Our calculations suggested that even for a short-period (SiC)_2_/(GeC)_2_ SL, the optical phonons are very well confined. As the bulk acoustical modes of GeC and SiC materials are similar (except for the difference in their frequencies), the SL vibrational modes (cf. Figure 5a) derived from the bulk materials are seen as a mixture of the two sets of acoustical phonon branches. 

Again, our simulations of $\omega_{j}^{SL}\left(\vec{k}\right)$ in SiC/GeC SLs confirmed that the folded acoustic phonons (see Figure 5a) appear below ω < 300 cm^−1^ which is a common overlapping acoustic mode region of the SiC–GeC bulk materials. Other high-frequency $\omega_{LA}$ acoustic modes falling in the frequency range of 470 cm^−1^ < ω < 620 cm^−1^, behave as the confined acoustic modes (CAMs) in the SiC layer. The RIM results reported here are in good agreement with those calculated by using an LCM approach [51].

##### [B] (SiC)_n_/(SnC)_n_

The results of RIM phonon dispersions $\omega_{j}^{SL}\left(\vec{k}\right)$ are displayed in Figure 6a along the Γ → Z direction for a (SiC)_2_/(SnC)_2_ [001] SL. The $\omega_{j}\left(\vec{k}\right)$ modes along the Γ → X direction for the bulk SiC (Figure 6b) and SnC (Figure 6c) materials are also shown by the red-, and blue-colored lines with phonon folding effects in the m-BZ using dotted lines. The dynamical properties clearly revealed atypically distinctive traits caused by the large mass difference between Si (28.08 amu) and Sn (118.7 amu) cations with respect to the common C (12.1 amu) anion. Unlike (SiC)_2_/(GeC)_2_ SLs, Figure 6b,c reveal that in (SiC)_2_/(SnC)_2_ SLs, the bulk $\omega_{LA}$ modes of SiC overlapping and even crossing the bulk SnC optical phonons. This means that the folded acoustic modes in (SiC)_m_/(SnC)_n_ SLs could become sensitive to the period of the superlattice.

Again, in the (SiC)_2_/(SnC)_2_ SL, the optical $(\omega_{LO}$−$\omega_{TO}$) vibrational modes of SiC-like (974–793 cm^−1^), and SnC-like (558–456 cm^−1^) phonon branches remained well separated and caused the confined optical modes (COMs). Due to a large (15%) difference in the lattice constants between SiC-SnC, one expects thin layer (SiC)_m_/(SnC)_n_ SLs to become mutually strained. Figure 6a, reveals a common bulk $\omega_{TA}$ like SiC- SnC acoustic phonons (ω < 300 cm^−1^) exhibiting highly dispersive mode behavior. A few non-dispersive $\omega_{LA}$-like SiC phonon modes (300 cm^−1^ < ω < 620 cm^−1^) are also noticed which are usually achieved in the COMs.

##### [C] (GeC)_n_/(SnC)_n_

The results of phonon dispersions $\omega_{j}^{SL}\left(\vec{k}\right)$ along the Γ → Z direction are displayed in Figure 7a for a (GeC)_2_/(SnC)_2_ [001] SL. The $\omega_{j}\left(\vec{k}\right)$ modes for bulk GeC (Figure 7b) and bulk SnC (Figure 7c) are also shown along the Γ → X direction using blue-, and red-colored lines, respectively, with the phonon folding effects in the m-BZ using dotted lines. Despite the difference in lattice constants (~10.5%) between GeC-SnC and large variations in the atomic masses of Ge (72.64 amu) and Sn (118.7 amu), the dynamical properties (see Figure 7a–c) of GeC/SnC SLs revealed similar behavior as seen in the LCM phonon dispersions $\omega_{j}^{SL}\left(\vec{k}\right)$ for the GaN/InN SLs [76].

Unlike (SiC)_2_/(SnC)_2_, the bulk optical phonon modes $(\omega_{LO}$−$\omega_{TO}$) of SnC (558–456 cm^−1^) (see Figure 3a,b and Figure 7c) do not overlap with those of the bulk GeC (749–626 cm^−1^) optical modes (see Figure 7b). Moreover, the acoustic phonon branches appear in the frequency range of ω < 370 cm^−1^—far below the SL optical modes. Again, Figure 7a reveals the common bulk $\omega_{TA}$-like GeC-SnC acoustic phonons below ω < 220 cm^−1^ exhibiting a highly dispersive behavior. Moreover, we observed non-dispersive $\omega_{LA}$-like GeC mode between 220 cm^−1^ < ω < 350 cm^−1^. These characteristics of mode behavior are generally associated with the COMs of SLs. Our RIM results are in qualitatively good agreement with those of the LCM calculations [51].

Detailed simulations exhibiting these, and many other important phonon features will be clarified in Section 3.2.3. Comprehensive RIM simulations of $\omega_{j}^{SL}\left(\vec{k}\right)$ in SLs with $k_{\left|\right|}\;and\;k_{⊥}$ to the growth direction are shown mixing with the acoustic phonons causing stop bands and anisotropy of phonons $\omega_{j}^{SL}\left(\vec{k}\right)$ in C-based SLs. Earlier, the minigap formation due to zone-folding in conventional GaAs/AlAs SLs was detected [77,78] and played an important role in reducing the phonon thermal conductivity [93,94].

#### 3.2.3. Acoustic Stopbands and Anisotropic Phonon Behavior

In designing electronic devices, one needs semiconductor materials of high thermal conductivity to remove the excess heat as fast as possible. In the conventional Si/Ge and GaAs/AlAs SLs, most theoretical analyses of heat conduction have employed the traditional Boltzmann transport equation (BTE) where the phonons are treated as particles, ignoring wave-like characteristics [93,94,95,96,97]. Few researchers, however, took alternative approaches explaining the reduction in thermal conductivity using phonon dispersions in SLs [88,89]. Whether thermal transport is dominated by particle motion, or wave propagation depends on the ratio of the SL period to the phonon mean free path. The crossover between these two characteristic regimes was studied earlier in terms of a simple lattice dynamical model [94]. In Section 3.2.3 [A]–[C], we presented our comprehensive results of $\omega_{j}^{SL}\left(\vec{k}\right)$ for (SiC)_n_/(GeC)_n_ SLs.

##### [A] (SiC)_n_/(GeC)_n_ SL Phonons in Γ → Z Direction

**Figure 8:**
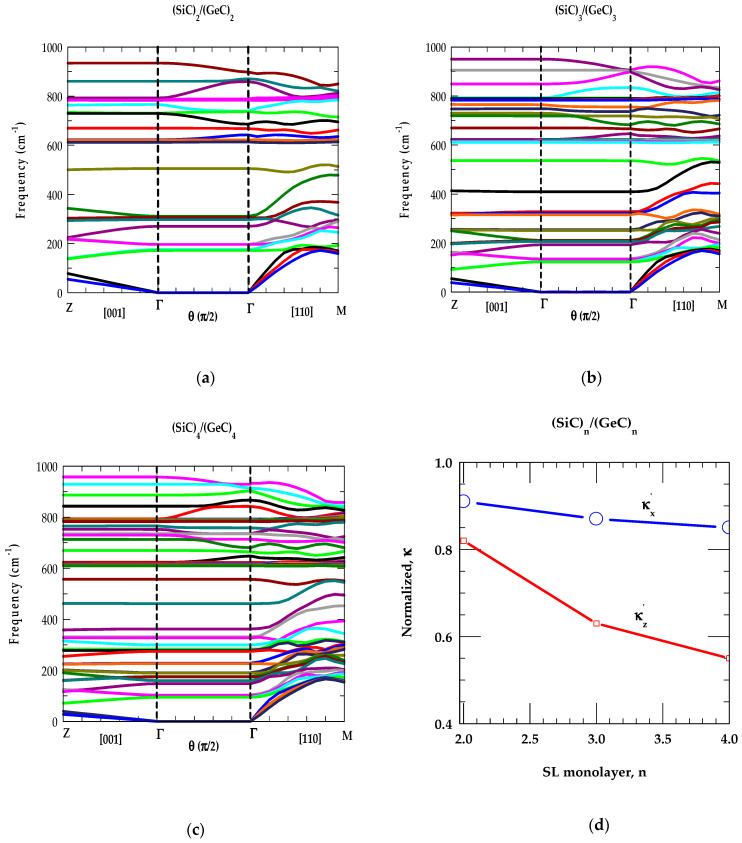
(**a**) Phonon dispersion curves $\omega_{j}^{SL}\left(\vec{k}\right)$ of a ${\left(SiC\right)}_{2}/({GeC)}_{2}$ SL for selected sets of wave vectors in the m-BZ. Left-hand-side of the figure represents $\omega_{j}^{SL}\left(\vec{k}\right)$ for |${\vec{k}}_{SL}$| parallel to the growth axis (Γ →Z), the right-hand side $\omega_{j}^{SL}\left(\vec{k}\right)$ for |${\vec{k}}_{SL}$| perpendicular (Γ →M) to the growth axis. The middle portion of the figure represents $\omega_{j}^{SL}\left(\vec{k}\right)$ for |${\vec{k}}_{SL}|$ = 0 (Γ → Γ) measured in terms of θ from growth direction it from 0→π/2 as $\left|{\vec{k}}_{SL}\right|$ goes from [001] to [100] in a plane normal to [010]. (**b**) same key as of Figure 8a but for ${\left(SiC\right)}_{3}/({GeC)}_{3}$ SL. (**c**) same key as of Figure 8a but for ${\left(SiC\right)}_{4}/({GeC)}_{4}$. (**d**) Normalized thermal conductivity, κ of ${\left(SiC\right)}_{n}/({GeC)}_{n}$ as a function of n (see text).

In the left panels of Figure 8a–c, we displayed our calculated RIM results (n = 2, 3, 4) of phonon dispersions $\omega_{j}^{SL}\left(\vec{k}\right)$ for the ${\left(SiC\right)}_{n}/({GeC)}_{n}$ SLs with wavevectors parallel $k_{\left|\right|}\;(or\;|{\vec{k}}_{SL}|=k_{z})$ to the growth [001] direction (i.e., from Γ→ Z). 

One must note that we chose z axis ([001]) as the growth direction of SLs. The x [100] and y [010] axes are chosen in directions with wave-vectors perpendicular $k_{⊥}\;\left(or\;\left|{\vec{k}}_{SL}\right|=k_{x},k_{y}\right)$ to the growth directions, respectively. If one focuses on the (SiC)_2_/(GeC)_2_ SL (say), the dispersions (see Figure 8a) along Γ → Z ($\;for\;k_{\left|\right|}$) and Γ → M directions (for $k_{⊥}$) are generated numerically following the method described in Section 2.2. Since the (2 × 2) SL contains two-unit cells each of bulk SiC and GeC materials, arranged along the growth direction [001], the SL m-BZ in the z direction is one-fourth, as far from the center as it is in the in-plane directions x [100] and y [010]. Thus, each of the four branches of the bulk material is folded back four times along the Γ → Z, which can most easily be seen for the longitudinal acoustic modes in the left panel of Figure 8a. 

Since the bulk optical modes of SiC and GeC have no frequencies in common; therefore, these modes are localized. This leads to a flat SL dispersion of the optical modes with localization of SiC optical phonons in the SiC layer. The GeC optical modes, on the other hand, are not localized, because they overlap with the $\omega_{LA}$ mode of the bulk SiC (see Figure 2a along the Γ → X direction in the BZ). Due to the non-analyticity of the Coulomb interaction as the SL |${\vec{k}}_{SL}$| → 0 in the m-BZ, the phonons ω(|${\vec{k}}_{SL}$| → 0) differ from Γ → Z (left panel) and Γ → M (right panel) directions, decoupling completely the transverse modes (see Figure 8a) in the Γ → M direction.

Except for a few overlapping GeC- and SiC-like low-frequency modes ω < 350 cm^−1^ in the Γ → Z direction, the calculations revealed $\omega_{LA}$ phonons of SiC falling in the GeC optical modes region 350 cm^−1^ < ω < 625 cm^−1^. High-frequency optical ($\omega_{TO(\Gamma )}and\omega_{LO(\Gamma )})$ modes occurring between 626 cm^−1^–748 cm^−1^ (GeC) and 793 cm^−1^–974 cm^−1^ (SiC) are well separated. Moreover, the results of $\omega_{j}^{SL}\left(\vec{k}\right)$ confirmed the low-frequency modes below ω < 167 cm^−1^ and those between ~350 cm^−1^ >ω > 167 cm^−1^ constituting the mixture of GeC-and SiC-like CAMs. Again, in ${\left(SiC\right)}_{n}/({GeC)}_{n}\;SLs$ the optical modes are confined in their respective GeC and SiC layers. Interestingly, $\omega_{LA}$ phonon continua of SiC with frequencies slightly <626 cm^−1^ and >350 cm^−1^ ${(i.e.,the\;\omega }_{LA}$ continuum of GeC) exhibited features like those of the GeC- and SiC-like COMs. We designated, however, these phonons as SiC-like confined modes, linked to the bulk $\omega_{LA}$ acoustic phonons of the binary SiC material.

##### [B] (SiC)_n_/(GeC)_n_ SL Phonons in Γ→ Γ Direction

In the middle panels of Figure 8a–c, we displayed our results of angular dependent $\omega_{j}^{SL}$ at the zone center $\left(\left|{\vec{k}}_{SL}\right|=0\right)$ along the Γ-Γdirection of ${\left(SiC\right)}_{n}/({GeC)}_{n}\;SLs$. The variation of θ from 0→π/2 is measured using the wavevector ${\vec{k}}_{SL}\;from$ the growth direction changing its value from [001] to [100] direction in the plane normal to [010]. Here, we observed two sets of optical vibrational modes: one associated with GeC-like (lower frequency set, between 626–748 cm^−1^) and the other linked to SiC-like modes (higher-frequency set, between 793–974 cm^−1^). Phonons with ω < 626 cm^−1^ are related to the acoustical vibrations. Consistent with our group-theoretic arguments, it is clearly observed that the results of angular dependent $\omega_{j}^{SL}\left(\vec{k}\right)$ for optical phonons in ${\left(SiC\right)}_{n}/({GeC)}_{n}\;SLs$ exhibited modes with either large, weak and/or zero angular dependencies. The observations are found in qualitatively good agreement with those of Refs. [74,75] for the conventional GaAs/AlAs SLs.

Our phonon dispersions for low-frequency acoustic modes $\omega_{j}^{SL}\left(\vec{k}\right)$ of ${\left(SiC\right)}_{n}/({GeC)}_{n}$ SLs revealed no angular dependency even if they are IR active. This is simply because the dipole moments induced by IR active acoustic phonons are typically very small. Another important indication of this study is the anisotropic dependency for the SL phonons at $\left|{\vec{k}}_{SL}\right|$ = 0 where the phonon frequencies remain unchanged for $\left|{\vec{k}}_{SL}\right|$ approaching zero from different in-plane directions.

##### [C] (SiC)_n_/(GeC)_n_ SL Phonons in Γ→ M Direction

In the right-side panels of Figure 8a–c, we displayed our simulated RIM results of $\omega_{j}^{SL}\left(\vec{k}\right)\;for{\;\left(SiC\right)}_{n}/({GeC)}_{n}\;SLs$ with wavevector $\left|{\vec{k}}_{SL}\right|$ perpendicular (i.e., in-plane Γ → M) to the growth direction. Once again, the phonon modes with ω higher (lower) than 626 cm^−1^ are derived from the optical (acoustical) branches of the SiC-GeC bulk materials. Due to large differences in the masses of constituent Si (28.1 amu), Ge (72.64 amu) cations with respect to common C (12.1 amu) anion, the folded acoustic phonon gaps in the growth direction (i.e., at Γ-point and edge Z-point) are seen much larger and flatter compared to the acoustic phonon gaps in the in-plane direction. As the degeneracies of transverse [$\omega_{TO}\left(\omega_{TA}\right)]$ modes are lifted in the Γ → M direction, the SL optical (acoustical) phonons, exhibited a strong mixture of $\omega_{TO}$, $\omega_{LO}$ $(\omega_{TA}$, $\omega_{LA})$ GeC-, and SiC-like modes due to spatial confinement and zone-folding effects. Thus, the study revealed complicated phonon dispersion curves with the appearance of several acoustic stop bands at certain finite values of wavevectors $\left|{\vec{k}}_{SL}\right|$. Earlier, in conventional GaAs/AlAs SLs, the stop bands with the mixing of acoustic phonons are observed experimentally [77] at oblique incidence and studied theoretically by using an elastic theory [78].

In Figure 8d, we reported our simulated results of normalized $\kappa_{x}^{\prime }\;and\;\kappa_{z}^{\prime }$ at 297 K for ${\left(SiC\right)}_{n}/({GeC)}_{n}$ as a function of n. Obviously, our comprehensive studies of phonon dispersions $\omega_{j}^{SL}\left(\vec{k}\right)\;$(cf. Section 3.2.3 [A], [B] and [C]) $of{\;\left(SiC\right)}_{n}/({GeC)}_{n}\;SLs$ have played crucial roles in simulating, κ. These results exhibited frequencies and phonon structures associated with the high-symmetry critical points at Γ, Z, and M which determine their role in simulating the complicated density of states at frequencies revealing the large phonon peaks with smaller values of group velocities $v_{gj}$ (Equation (6)). By using Equations (5) and (6) and $\omega_{j}^{SL}\left(\vec{k}\right)$, we have calculated the normalized phonon conductivity $\kappa^{\prime }$ $\;\left(\equiv \frac{\kappa_{SL}}{\kappa_{avg}};\kappa_{x}^{\prime },\kappa_{z}^{\prime }\right)$ in the ideal short-period ${\left(SiC\right)}_{n}/({GeC)}_{n}$ SLs for different, n. According to the Fourier’s law, the $\kappa_{avg}$ values are obtained $\frac{\left(\kappa_{XC}+\kappa_{YC}\right)}{2}\;and$ $\frac{\kappa_{XC}.\kappa_{YC}}{\left(\kappa_{XC}+\kappa_{YC}\right)}$ for in-plane and cross-plane directions, respectively.

**Figure 9:**
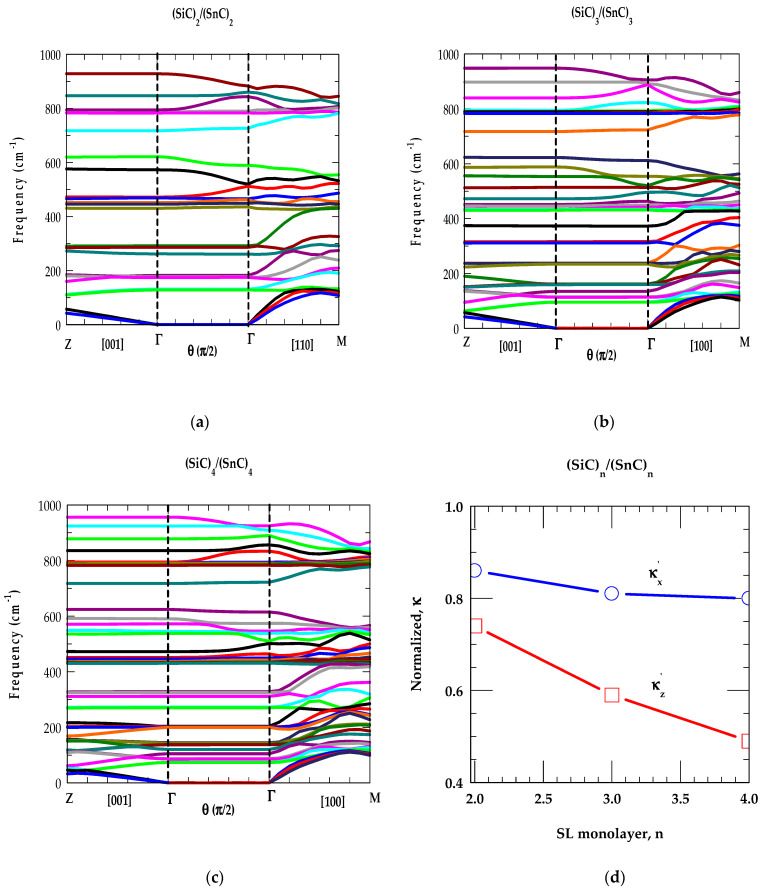
(**a**) Phonon dispersion curves $\omega_{j}^{SL}\left(\vec{k}\right)$ of a ${\left(SiC\right)}_{2}/({SnC)}_{2}$ SL for selected sets of wave vectors in the m-BZ. Left-hand-side of the figure represents $\omega_{j}^{SL}\left(\vec{k}\right)$ for |${\vec{k}}_{SL}$| parallel to the growth axis (Γ →Z), the right-hand side $\omega_{j}^{SL}\left(\vec{k}\right)$ for |${\vec{k}}_{SL}$| perpendicular (Γ →M) to the growth axis. The middle portion of the figure represents $\omega_{j}^{SL}\left(\vec{k}\right)$ for |${\vec{k}}_{SL}|$ = 0 (Γ → Γ) measured in terms of θ from growth direction it from 0→π/2 as $\left|{\vec{k}}_{SL}\right|$ goes from [001] to [100] in a plane normal to [010]. (**b**) same key as of Figure 8a but for ${\left(SiC\right)}_{3}/({SnC)}_{3}$ SL. (**c**) same key as of Figure 8a but for ${\left(SiC\right)}_{4}/({SnC)}_{4}$. (**d**) Normalized thermal conductivity, κ of ${\left(SiC\right)}_{n}/({SnC)}_{n}$ as a function of n (see text).

**Figure 10:**
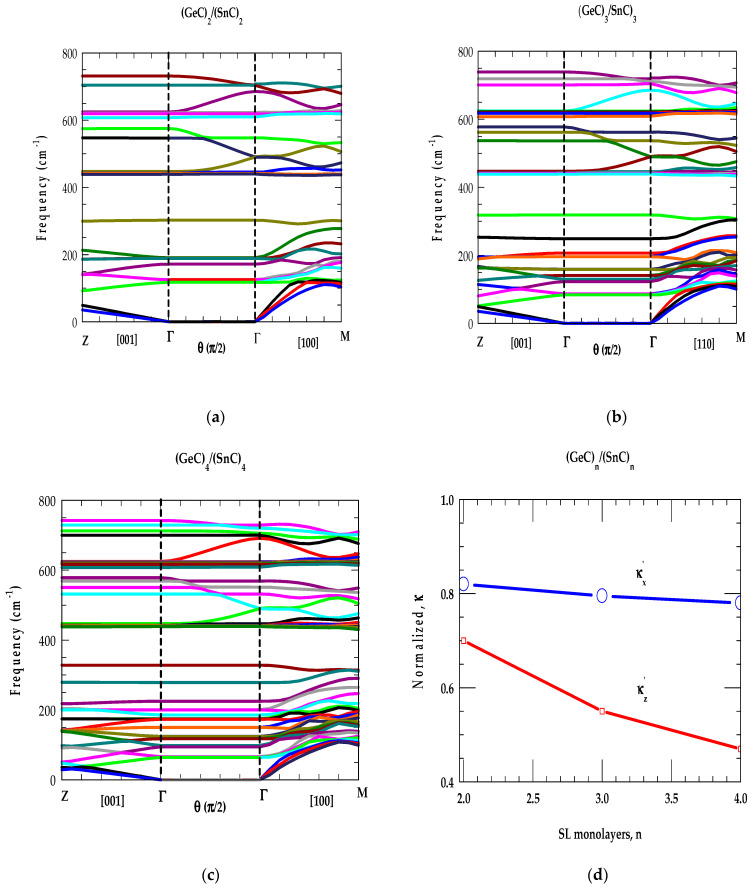
(**a**) Phonon dispersion curves $\omega_{j}^{SL}\left(\vec{k}\right)$ of a ${\left(GeiC\right)}_{2}/({SnC)}_{2}$ SL for selected sets of wave vectors in the m-BZ. Left-hand-side of the figure represents $\omega_{j}^{SL}\left(\vec{k}\right)$ for |${\vec{k}}_{SL}$| parallel to the growth axis (Γ →Z), the right-hand side $\omega_{j}^{SL}\left(\vec{k}\right)$ for |${\vec{k}}_{SL}$| perpendicular (Γ →M) to the growth axis. The middle portion of the figure represents $\omega_{j}^{SL}\left(\vec{k}\right)$ for |${\vec{k}}_{SL}|$ = 0 (Γ → Γ) measured in terms of θ from growth direction it from 0→π/2 as $\left|{\vec{k}}_{SL}\right|$ goes from [001] to [100] in a plane normal to [010]. (**b**) same key as of Figure 8a but for ${\left(GeC\right)}_{3}/({SnC)}_{3}$ SL. (**c**) same key as of Figure 8a but for ${\left(GeC\right)}_{4}/({SnC)}_{4}$. (**d**) Normalized thermal conductivity, κ of ${\left(GeC\right)}_{n}/({SnC)}_{n}$ as a function of n (see text).

Without considering the relaxation-time effects associated with phonon scattering from interfaces, defects, and surface roughness, we reported our simulated results in Figure 8d for the normalized κ’ values of ${\left(SiC\right)}_{n}/({GeC)}_{n}$ as a function of n. The main characteristics of these results are in good agreement with those reported by using an optical pump-and-probe technique for the conventional GaAs/AlAs SLs [93]. Similar calculations are also performed for ${\left(SiC\right)}_{n}/({SnC)}_{n},\;{and\;\left(GeC\right)}_{n}/({SnC)}_{n}$ SLs and the results are shown in Figure 9a–d, and Figure 10a–d, respectively.

The results of our study on short-period SLs (n = 2, 3, 4) clearly suggest that the phonon-gap and anti-crossing induced reduction in $v_{gi}$ of the acoustic modes contribute less to superlattices than they contribute to the bulk materials. Besides the mode-specific heat $C_{ph}$($\omega_{j}^{SL}$) and phonon group velocity, the atomic mass mismatch (ratio) of the constituent atoms in the SL also played an important role in the phonon conductivities (cf. Figure 8d, Figure 9d and Figure 10d). Our results should be viewed as complementary to the existing experimental and theoretical results reported by others in the conventional GaAs/AlAs and Si/Ge SLs [93,94,95,96,97]. From an application standpoint, if low (high) thermal conductivity is desired, as in thermoelectric (high dissipation) materials, the designing of a SL with a large (small) mass mismatch (ratio) between the constituent atoms would be beneficial. 

## 4. Conclusions and Future Development Directions

By using the short-range interactions of bulk materials and meticulously treating the long-range Coulomb interactions in the framework of a realistic RIM [63], we reported the results of our systematic phonon dispersion $\omega_{j}^{SL}\left(\vec{k}\right)$ calculations $forthenovelshort-period{\left(XC\right)}_{m}/({YC)}_{n}\;\left[001\right]\;SLs\;$(m = n = 2, 3, 4). Unlike the conventional GaAs-AlAs system, where the heavier common As-anion and lighter Ga- or Al-cations caused well separated optical phonons between GaAs, AlAs with gaps in their one-phonon density of states [49,50,51], the situation is quite different in the XC-YC materials. Due to lighter common C-anion and heavier X- and/or Y-cations, the RIM phonon dispersions revealed well-separated optical phonons between XC, YC with a wider phonon gap in YC than XC [46]. By varying the period of SL and choosing the appropriate wavevectors $\left|{\vec{k}}_{SL}\right|$(along the growth $k_{\left|\right|}$ and in-plane $k_{⊥}$ directions), our quantitative analyses of phonon dispersions $\omega_{j}^{SL}\left(\vec{k}\right)$ in (XC)_m_/(YC)_n_ yielded valuable information. Due to zone-folding effects, the in-plane modes revealed complicated phonon dispersion curves causing anisotropy of phonons with a strong mixture of low energy $\omega_{TA}$, $\omega_{LA}$ XC-like, YC-like modes causing stop bands at certain finite values of wavevectors $\left|{\vec{k}}_{SL}\right|$ in short-period SLs. As the degeneracies of transverse modes are lifted, each folded band exhibits zero velocity at the zone boundaries and zone center. This anisotropy and mixing of phonons coupled with zero velocities at the zone boundary and zone center modes in (XC)_m_/(YC)_n_ SLs caused significant modifications in the thermal properties via changes in their phonon velocities, specific heat, and density of states [54,55,56].

By considering perfect interfaces in an ideal short-period (XC)_n_/(YC)_n_ SLs, the RIM results of normalized phonon thermal conductivities $\kappa^{\prime }\left(\equiv \frac{\kappa_{SL}}{\kappa_{avg}};\kappa_{z}^{\prime },\kappa_{x}^{\prime }\right)$ are reported in the growth [001] and in-plane [100] directions using different values of n. In the framework of a constant relaxation time approximation, we followed the earlier methodologies for simulating the phonon thermal conductivities [93,94,95] by including contributions from the acoustic and optical phonons. Consistent with the conventional SLs, our results revealed an increase in the normalized values of $\kappa_{z}^{\prime },\kappa_{x}^{\prime }$ with the decrease in n. The in-plane $\kappa_{x}^{\prime }$ value is higher than $\kappa_{z}^{\prime }$ because the vibrational separation has a smaller effect on the in-plane phonon propagation. The outcome of our study is in good agreement with those reported by others [93,94,95]. From the physics standpoint, it simply means that the heat is being transferred more efficiently through the material as interfaces between the layers become more frequent, leading to a greater number of phonons scattering events. This perspective, under certain conditions, results in a net increase in thermal conductivity by decreasing n. For n > 2, the decrease in $\kappa^{\prime }$ in C-based SLs is mainly caused by the decrease in phonon group velocities. To focus on such low-energy phonons, it is essential to develop non-destructive surface imaging techniques by utilizing orientation-dependent phonon properties in C-based and other technologically important nanomaterials.

To understand the phonon-mediated thermal transport in SLs, the choice of first-principles calculations is complicated both by the competing effects of lattice anharmonicity and electron correlation effects [102]. Analysis of the harmonic and anharmonic properties depends on the precise computation of interatomic forces, which strongly differs in the ability to accurately describe the electronic structure of materials. While different methods to improve the electronic structure calculations in strongly electron-correlated systems are available, the implications of these methods on the accuracy of thermal transport predictions are unknown [102]. Again, the impact of point defects, impurities, and grain boundaries resulting in additional phonon scattering for causing a reduction in the thermal conductivities has also been investigated [85,86,87,88,89,90,91,92]. However, there still exist many discussions over the anisotropic nature of the calculated thermal transport in different SLs. In strained layer SLs, one could also expect rough interfaces between the XC-YC constituent layers. A few highly computationally intensive studies based on the Boltzmann equations and molecular dynamics simulations [95,96] have suggested that interface roughness scattering plays an important role in explaining the experimental reduction in the thermal conductivity observed in Si/Ge SLs. In our lattice dynamical approach, while we did not include the influence of surface roughness for investigating $\kappa^{\prime }$, it should be considered in future lattice dynamical calculations.

To explore the unique thermal transport properties in nanomaterials, several experimental techniques have been developed in recent years [103,104]. These intensive efforts are primarily adopted for nanotubes, nanowires, and nanoribbons. We expect that such attempts, if extended to SLs, will help comprehend not only the fundamental understanding of thermal physics in LDHs but could also shine light on their use in broad industrial applications including thermal management of modern electronics, nanostructure-based thermoelectric, and thermal interface materials. In conclusion, the systematic and rationalized study reported here provides a rigorous description of the underlying mechanisms that govern phonon transport in technologically relevant C-based XC/YC SLs. This cost-effective method can be extended to a wide variety of LDH nanostructures, and we feel that the results reported here will certainly be helpful in designing SL-based device structures with tailored thermal properties for use in future nano-/micro-scale devices for energy and biomedical application needs.

## Figures and Tables

**Table 1:** For zb XC (SiC, GeC and SnC) materials, the simulated results of rigid-ion-model (RIM) phonon frequencies (in cm^−1^) at high critical points Γ, X, and L are compared with the existing experimental (Refs. [45,46]) and ab initio calculations (Refs. [53,59,60]). The optical phonon splitting ${∆\omega }^{opt}\left(\equiv \omega_{LO\left(\Gamma \right)}-\omega_{TO\left(\Gamma \right)}\right)$ is also compared with (Refs. [45,46,53,59,60]) experimental and theoretical data (see: text).

Material	$\omega_{LO(\Gamma )}$	$\omega_{TO(\Gamma )}$	$\omega_{LO(X)}$	$\omega_{TO(X)}$	$\omega_{LA(X)}$	$\omega_{TA(X)}$	$\omega_{LO(L)}$	$\omega_{TO(L)}$	$\omega_{LA(L)}$	$\omega_{TA(L)}$	${∆\omega }^{opt}$
3C-SiC ^a)^	974	797	828	760	639	373	857	787	591	250	177
Others	974 ^b)^	793 ^b)^	830 ^b)^	759 ^b)^	644 ^b)^	373 ^b)^	850 ^b)^	770 ^b)^	605 ^b)^	260 ^b)^	181 ^b)^
	972 ^c)^	796 ^c)^	829 ^c)^	761 ^c)^	640 ^c)^	373 ^c)^	838 ^c)^	766 ^c)^	610 ^c)^	266 ^c)^	176 ^c)^
	953 ^d)^	783 ^d)^	811 ^d)^	749 ^d)^	623 ^d)^	364 ^d)^	832 ^d)^	755 ^d)^	608 ^d)^	260 ^d)^	170 ^d)^
	945 ^e)^	774 ^e)^	807 ^e)^	741 ^e)^	622 ^e)^	361 ^e)^	817 ^e)^	747 ^e)^	601 ^e)^	257 ^e)^	171 ^e)^
	956 ^f)^	783 ^f)^	829 ^f)^	755 ^f)^	629 ^f)^	366 ^f)^	838 ^f)^	766 ^f)^	610 ^f)^	261 ^f)^	173 ^f)^
											
GeC ^a)^	749	626	697	617	348	211	705	621	326	166	123
Others	748 ^f)^	626 ^f)^	697 ^f)^	617 ^f)^	348 ^f)^	214 ^f)^	705 ^f)^	612 ^f)^	331 ^f)^	162 ^f)^	122 ^f)^
SnC ^a)^	558	456	512	454	216	141	524	454	214	102	102
Others	558 ^f)^	456 ^f)^	503 ^f)^	450 ^f)^	216 ^f)^	134 ^f)^	516 ^f)^	440 ^f)^	199 ^f)^	109 ^f)^	102 ^f)^

^a)^ Our; ^b)^ Ref. [46]; ^c)^ Ref. [45]; ^d)^ Ref. [59]; ^e)^ Ref. [60]; ^f)^ Ref. [53].

## Data Availability

The data that support the findings of this study are available from the corresponding author upon reasonable request.
